# Activated Screen-Printed Boron-Doped Diamond Electrode for Rapid and Highly Sensitive Determination of Curcumin in Food Products

**DOI:** 10.3390/ma16216826

**Published:** 2023-10-24

**Authors:** Jędrzej Kozak, Katarzyna Tyszczuk-Rotko, Aleksy Keller, Magdalena Wójciak, Ireneusz Sowa

**Affiliations:** 1 Faculty of Chemistry, Institute of Chemical Sciences, Maria Curie-Skłodowska University in Lublin, 20-031 Lublin, Poland; 2 Department of Analytical Chemistry, Medical University of Lublin, 20-093 Lublin, Poland

**Keywords:** curcumin, polyphenols, antioxidants, screen-printed sensor, stripping voltammetry, food analysis

## Abstract

Due to a great interest in the beneficial properties of polyphenolic antioxidant curcumin (CCM), sensitive and accurate methods for determining CCM are needed. The purpose of our research was to develop a very simple, fast, and sensitive differential pulse adsorptive stripping voltammetric (DPAdSV) procedure using an electrochemically activated screen-printed boron-doped diamond electrode (aSPBDDE) for the determination of CCM. The activation of the SPBDDE was accomplished in a solution of 0.1 mol/L NaOH by performing five cyclic voltammetric scans in the range of 0–2 V, at ν of 100 mV/s. The changes in surface morphology and the reduction of the charge transfer resistance due to the activation of the electrode resulted in the amplification of the CCM analytical signal on the aSPBDDE. As a result, an extremely sensitive measurement tool was formed, which under optimized conditions (0.025 mol/L PBS of pH = 2.6, E_acc_ of 0.3 V, t_acc_ of 90 s, ΔE_A_ of 100 mV, ν of 150 mV/s, and t_m_ of 10 ms) allowed us to obtain a limit of detection (LOD) of 5.0 × 10^−13^ mol/L. The aSPBDDE has proven to be a highly effective tool for the direct determination of CCM in food samples with high accuracy and precision. The results are in agreement with those obtained using ultra-high-performance liquid chromatography coupled with mass spectrometry and electrospray ionization (UHPLC-ESI/MS).

## 1. Introduction

Curcumin (CCM) is a yellow polyphenolic compound obtained from the root of the Curcuma longa (turmeric) plant. CCM has a wide range of biological actions and pharmacological effects. These properties include the antioxidant effect, which consists in curcumin inhibiting the formation of free radicals in the blood and tissues. This compound is also characterized by anti-inflammatory, anti-cancer, antimicrobial, and immunomodulatory effects [1,2,3]. CCM is also used in the treatment of malaria, diabetes, arthritis, skin diseases, and cardiovascular diseases, as well as diseases related to the nervous system, such as dementia, Alzheimer’s, or multiple sclerosis. In addition, CCM is considered a safe substance; it does not show toxic, teratogenic, or genotoxic effects. On the other hand, too high doses, exceeding 12 g per day, may result in health problems such as diarrhoea, nausea, and chronic abdominal pain [1,4].

Due to its intense yellow colour, CCM is a commonly used food additive, both colouring and spicing, e.g., in mustard, curry, margarine, sweets, and soft drinks [5,6]. Depending on the type of food products, the amount of added CCM ranges from 5 to 500 mg kg^−1^. CCM is also used in dyeing fabrics [1]. Bearing in mind the multitude of benefits of curcumin consumption, it is currently widely used to produce various types of supplements and functional foods, i.e., foods that bring health benefits resulting from the active ingredients present in them when consumed. In the case of curcumin, a problem may be its poor solubility in water; therefore, to increase its bioavailability, it is dispersed in emulsified systems or encapsulated [7].

Due to the great interest in the beneficial properties of CCM and, consequently, the growing number of studies on this compound, sensitive and accurate methods for determining CCM are needed. So far, a number of methods have been used in the determination of CCM, such as capillary electrophoresis (CE) [8], spectrofluorimetry [9], spectrophotometry [10], high-performance liquid chromatography (HPLC) [11], liquid chromatography coupled with tandem mass spectrometry (LC-MS/MS), or resonance light scattering (RLS) [12,13]. Electrochemical methods characterized by low cost, fast response, and simplicity of operation, as well as high selectivity and sensitivity, are also very popular [14]. Numerous voltammetric procedures for the determination of CCM [1,2,3,4,5,15,16,17,18,19] can be found in the literature, but they only allow obtaining LODs of the order of 10^−8^–10^−10^ mol/L. To our knowledge, the lowest limit of detection (LOD: 2.0 × 10^−13^ mol/L) was obtained using the voltammetric procedure described in publication [20]; however, the sensor (NSrGO/Ru@AuNPs/GCE) used requires a time-consuming, multi-step preparation. It should be added that low detection and quantification limits allow not only for the determination of trace concentrations of analyte but also high dilutions of the analyzed samples, thus minimizing interferences for the sample matrix. Therefore, the development of new procedures with better analytical parameters than those described in the literature is advisable. A comparison of the different CCM determination methods is shown in Table 1.

Among the ever-increasing number of electrochemical sensors being developed, screen-printed sensors are currently enjoying great interest. These miniature devices typically consist of a three-electrode array printed with conductive inks on a ceramic or plastic substrate [21,22]. Their undoubted advantages include low cost, compatibility with portable analyzers, the ability to determine various substances, and a multitude of materials that can be used for the production of electrodes. In addition, they have a low background current and are easily modifiable to suit different applications [23,24,25,26].

A popular electrode material used in the production of both conventional and screen-printed electrodes is boron-doped diamond (BDD). BDD is most often obtained by chemical vapour deposition, and its properties can be changed by changing the doping density, the ratio of sp^2^ to sp^3^ hybridized carbon, or the crystallographic orientation. The electrode made of BDD (BDDE) is characterized by a wide range of work potentials, chemical inertness and stability, and a long lifetime. BDDE polarization at high positive potentials results in a low electrochemical activity for the oxygen evolution reaction and a high chemical reactivity for organic oxidation. BDDE is also heat and pressure resistant as well as fouling resistant [27,28,29,30]. The inks used for the production of screen-printed sensors contain an organic binder, composed mainly of various types of resins and other additives, the presence of which results in a slowdown in the kinetics of electrochemical reactions. Electrochemical activation of the electrodes is aimed at removing the organic components of the ink; it can also result in an increase in the roughness and active surface of the working electrode, as well as its functionalization, thanks to which it significantly improves the parameters of the sensors used [31,32,33].

To our knowledge, there is no work in the literature describing the use of a screen-printed sensor in the analysis of CCM. Additionally, the oxidation peak potential of CCM, and the enhancement of rifampicin (RIF) analytical signals after the electrochemical activation of electrodes observed in earlier work, encouraged us toward the use of an electrochemically activated screen-printed boron-doped diamond electrode (aSPBDDE) in CCM analysis [33]. Therefore, the aim of this work was to develop a highly sensitive and accurate voltammetric procedure for the determination of CCM using an aSPBDDE.

## 2. Materials and Methods

### 2.1. Apparatus

Voltammetric studies were conducted using a μAutolab (Eco Chemie, Utrecht, The Netherlands) controlled by GPES 4.9 software. The same analyzer was also used to record differential capacity curves. In those cases, it was controlled by FRA 4.9 software. All measurements were performed in a standard 10 mL quartz cell with a screen-printed boron-doped diamond working electrode, a silver pseudo-reference electrode, and a carbon counter electrode (Metrohm-DropSens, Oviedo, Spain). Chromatographic analyses were performed on an ultra-high-performance liquid chromatograph (UHPLC) Infinity Series II with an Agilent 6224 ESI/TOF mass detector (Agilent Technologies, Santa Clara, CA, USA) with an RP18 reversed-phase column Titan (Supelco, Sigma-Aldrich, St. Louis, MO, USA) (10 cm × 2.1 mm i.d., 1.9 µm particle size). Microscopic images were obtained using a high-resolution scanning electron microscope Quanta 3D FEG (FEI, Hillsboro, OR, USA).

### 2.2. Reagents and Solutions

The weighted amount of CCM (Merck, Darmstadt, Germany, ≥80% CCM) was dissolved in ethanol, yielding a 10^−3^ mol/L stock solution of this compound. This solution was diluted with ethanol to obtain 10^−4^, 10^−5^, 10^−6^, and 10^−7^ mol/L solutions. In addition, 0.1 mol/L solutions of phosphate-buffered saline (PBS) with the following pH values were used to test the effect of pH on the CCM peak current: 2.6, 4.1, 4.9, 6.0, 7.1, and 8.0. The 10^−3^ mol/L solutions of glucose (≥99.5%), ascorbic acid (≥99%), Ca^2+^ (1 g/L), and Mg^2+^ (1 g/L) ions were made using reagents purchased from Merck. Acetonitrile and formic acid were MS grade (Merck, Darmstadt, Germany). All solutions were made with ultrapurified water (>18 MW cm, Millipore, Watford, UK). The analyzed food samples, i.e., turmeric, herbal tea, and immune shot, were purchased from a nearby supermarket or pharmacy (Lublin, Poland).

### 2.3. Sample Preparation

First, 0.2 g of turmeric powder (100% CCM, Wolka Kosowska, Poland) was extracted with 20 mL of ethanol for 1 h in an ultrasonic bath at 55 °C [34]. The resulting extract was filtered using a 0.22 µm Millipore syringe filter (Merck, Darmstadt, Germany). One bag of herbal tea (organic herbal tea with curcumin, 40% CCM, Grodzisk, Poland) was brewed with 200 mL of boiling water under cover for 15 min and then also filtered. The shot sample (immunity shot, 4% CCM, Warsaw, Poland) was only filtered before analysis.

### 2.4. Differential Pulse Adsorptive Stripping Voltammetric (DPAdSV) Analysis of CCM

**Figure 1:**
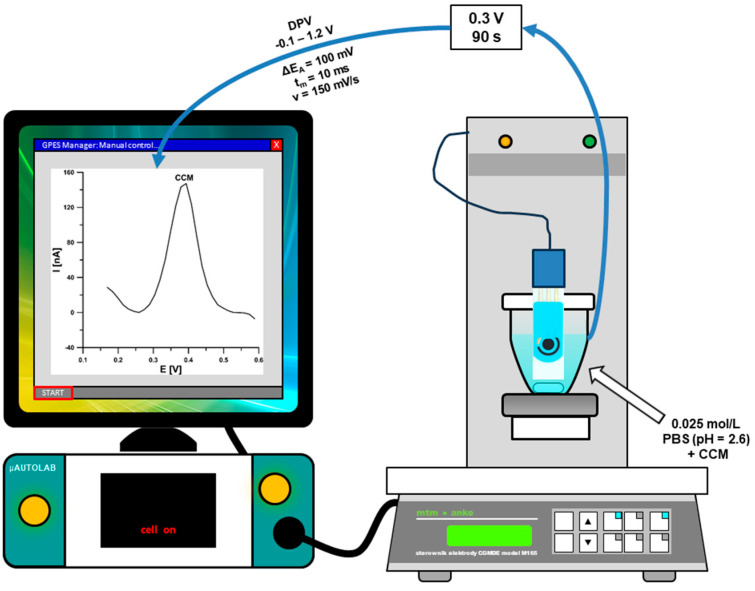
A diagram of the apparatus and the DPAdSV procedure used for the voltammetric determination of CCM.

DPAdSV analysis of CCM under optimized conditions was performed in 0.025 mol/L PBS (pH = 2.6). The determined compound had accumulated on the aSPBDDE surface at the potential of 0.3 V (E_acc._) for 90 s (t_acc._). Voltammetric curves were registered in a potential range from −0.1 to 1.2 V with an amplitude (ΔE_A_) of 100 mV, a modulation time t_m_ of 10 ms, and a scan rate (ν) of 150 mV/s. From each voltammogram the background curve was subtracted, and the baseline was corrected. A diagram of the apparatus and the DPAdSV procedure used for the voltammetric determination of CCM appears below (Figure 1).

### 2.5. UHPLC-ESI/MS Analysis

For chromatographic technique, the mobile phase composed of 45% acetonitrile in water with the addition of 0.05% formic acid at a flow rate of 0.2 mL/min was used. A thermostat temperature was set at 30 °C. Electrospray ionization with a negative mode (ESI-) was used for MS analysis with parameters as follows: drying gas temperature 325 °C, drying gas flow 8 L/min, nebulizer pressure 30 psi, and capillary voltage 3500 V; the skimmer and fragmentor voltage were 65 V and 200 V, respectively. Ions were acquired from 100 to 1200 *m*/*z*. Quantification was based on external calibration and a standard addition method.

## 3. Results and Discussion

### 3.1. Preliminary Studies

**Figure 2:**
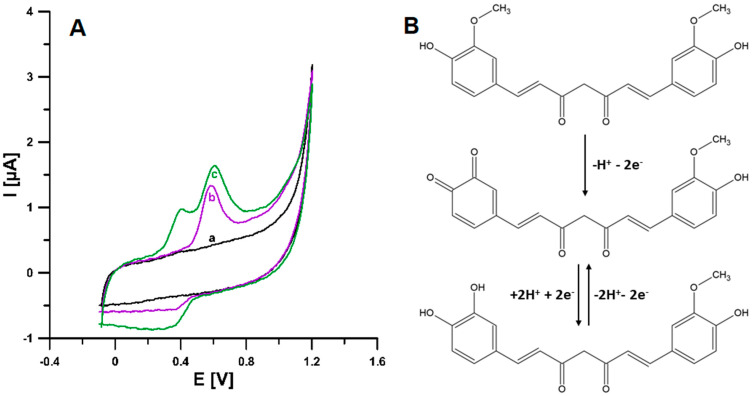
(**A**) CV curves obtained at the SPBDDE in the 0.1 mol/L HNO_3_ in the presence of 0 (a) and 2 × 10^−6^ mol/L (b—first cycle and c—subsequent cycles) CCM using a scan rate of 100 mV/s. (**B**) Mechanism of CCM oxidation.

Cyclic voltammetry was used to investigate the voltammetric response of the CCM on a screen-printed boron-doped diamond electrode (SPBDDE). Cyclic voltammograms were recorded for 2 × 10^−6^ mol/L CCM in 0.1 mol/L nitric acid, in the potential range from −0.1 to 1.2 V and at a ν of 100 mV/s (Figure 2A). In the first cycle (curve b), there is one oxidation peak at the potential of 0.60 V and one reduction peak at the potential of 0.31 V. In the subsequent cycles, the appearance of the second oxidation signal at the potential of 0.4 V was observed. As can be seen in Figure 2, the CCM peak on curve b is higher than the CCM peak on curve c. This difference is connected with the gradual oxidation of CCM in the second cycle. Based on the obtained curves, the oxidation of CCM on SPBDDE can be described as quasi-reversible. The source of the anodic peak at 0.6 V is the 2-electron and 1-proton oxidation reaction of the 3-methoxy-4-hydroxyphenyl substituent to o-benzoquinone. The appearance of the second anodic peak can be explained by the reaction of the o-benzoquinone substituent with the participation of 2 electrons and 2 protons with the formation of catechol [14,15] (Figure 2B). Due to the intensity of the signal in the later stages of the research, the focus was on the oxidation peak at the potential of 0.6 V.

**Figure 3:**
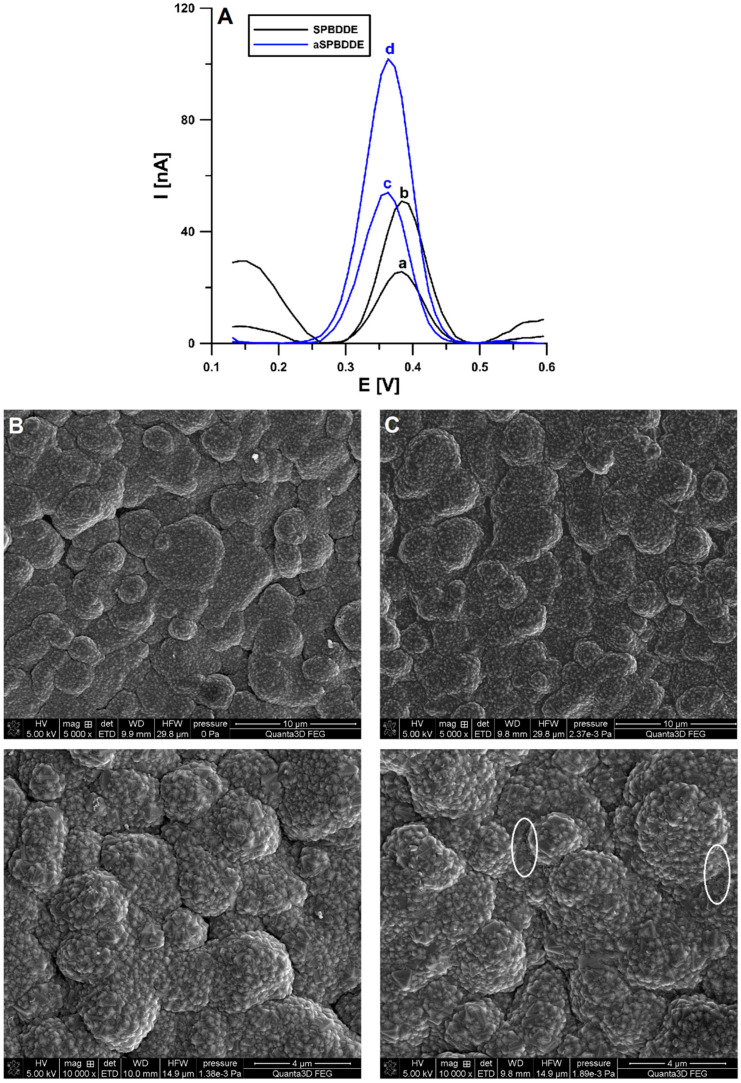
(**A**) Voltammograms of 1 × 10^−9^ (a and c) and 2 × 10^−9^ mol/L (b and d) CCM on SPBDDE (a and b) and aSPBDDE (c and d). The DPAdSV parameters: ν of 100 mV/s, t_m_ of 10 ms, ΔE_A_ of 100 mV, E_acc._ of 0 V, and t_acc._ of 60 s. SEM images of SPBDDE (**B**) and aSPBDDE (**C**).

In the later stage of the experiments, the current intensity of the CCM oxidation peaks on the SPBBDDE and on the same electrochemically activated electrode (aSPBDDE) were compared. Activation was performed in a solution of 0.1 mol/L NaOH by performing 5 CV scans in the range of 0–2 V, at ν of 100 mV/s. The method of electrode activation was not accidental. It was selected on the basis of previously conducted research [33]. As can be seen in Figure 3A, the peak currents of CCM obtained at the aSPBDDE are almost twice as high as on the non-activated electrode (50.1 vs. 26.0 nA for 1 × 10^−9^ mol/L CCM and 108.0 vs. 52.0 nA for 2 × 10^−9^ mol/L CCM, respectively). This result is connected with the fact that the electrochemical activation of SPBDDE affects its morphology and electrochemical properties, which may result in an increase in the analytical signals of the tested substances. A comprehensive characterization of these sensors (SPBDDE vs. a SPBDDE) using a number of techniques such as CV, electrochemical impedance spectroscopy (EIS), as well as optical profilometry and scanning electron microscopy (SEM), was performed as part of our previous research [33]. The sample SEM images exposing the morphological changes of an aSPBDDE surface are shown in Figure 3B,C. As a result of electrochemical activation, the organic binder is removed, which increases the number and size of pores near the electrode surface. In previous research [33], we found that the charge transfer resistance (R_ct_) is reduced from 286.5 to 105 Ω cm^2^ after the electrode activation. Moreover, optical profilometry showed an increase in surface roughness (0.451 vs. 0.517 µm) and total profile height (7.833 vs. 10.627 um). However, there was no change in the active surface of the working electrode (0.0146 ± 0.000510 vs. 0.0157 ± 0.000470 cm^2^).

### 3.2. Influence of pH and Concentration of Supporting Electrolyte

**Figure 4:**
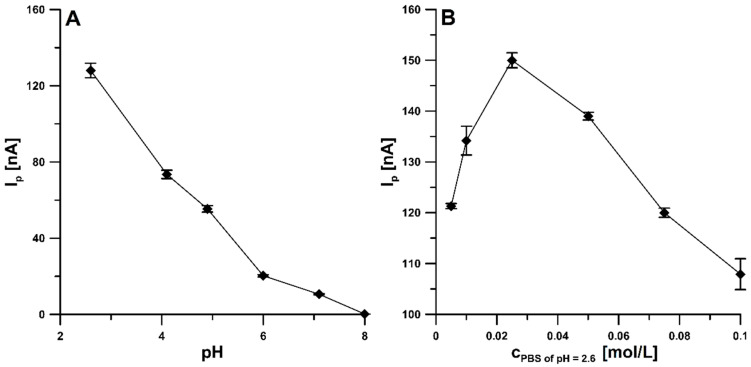
Effect of pH value (**A**) and concentration of the PBS of pH 2.6 (**B**) on CCM I_p_. The DPAdSV parameters: ν of 100 mV/s, t_m_ of 10 ms, ΔE_A_ of 100 mV, E_acc._ of 0 V, and t_acc._ of 60 s. The average values of the CCM peak current are presented with the standard deviation of n = 3.

In order to find the optimal pH value of the solution in which the experiments are carried out, the voltammetric response of 1 × 10^−9^ mol/L CCM in 0.1 mol/L PBS for pH in the range from 2.6 to 8.0 was tested. The peak current was demonstrated to decrease with an increase in the pH value. The highest peak was obtained in a buffer with a pH of 2.6; therefore, this value was chosen (Figure 4A). The decrease in CCM peak current with increasing pH value may be related to the changing stability of CCM molecules in the solution. According to the literature data, CCM is highly unstable under alkaline conditions due to the rapid hydrolytic degradation of feruolmethane and ferulic acid [4]. Moreover, with the increase in the solution’s pH, the potential of oxidation peaks of CCM negatively shifted. These results might suggest that protons were directly involved in the electrochemical oxidation of CCM.

Then, it was examined how the current intensity of the CCM peak changes at pH = 2.6 depending on the concentration of PBS, which was changed from 0.005 to 0.1 mol/L (Figure 4B). The CCM analytical signal increased with increasing electrolyte concentration and reached a maximum with the 0.025 mol/L PBS solution, so this concentration was implemented for further stages of research. The increase in the CCM peak current intensity is most likely related to the improvement of the electrical conductivity of the supporting electrolyte, while the decrease in the current intensity above the PBS concentration of 0.025 mol/L is related to the interactions in the solution that hinder the adsorption of the analyte onto the electrode surface.

### 3.3. Adsorption Studies

**Figure 5:**
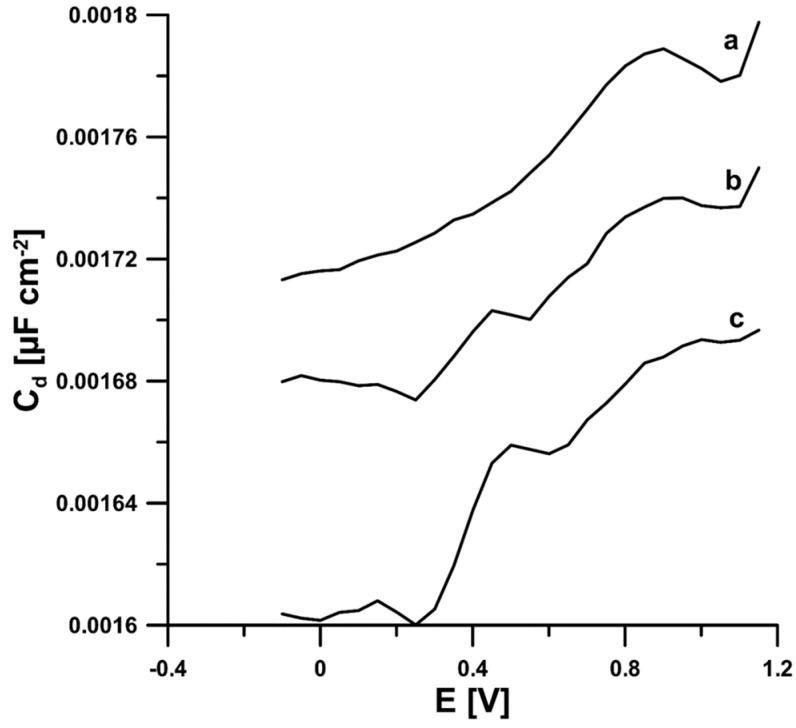
The differential capacity–potential curves of the double-layer interface aSPBDDE/PBS (pH 2.6) in the presence of 0 (a), 5 × 10^−6^ (b), and 1 × 10^−5^ (c) mol/L CCM.

In order to determine the nature of the CCM electro-oxidation process on the aSPBDDE, differential capacity–potential curves of the double-layer interface aSPBDDE/PBS of pH = 2.6 in the potential range of −0.1–1.2 V at a frequency of 200 Hz were recorded. On the obtained curves for 5 × 10^−6^ and 1 × 10^−5^ mol/L CCM, two desorption peaks were observed at the potentials of 0.15 and 0.48 V (Figure 5). As can be seen, the desorption signals increase with increasing CCM concentration. Their presence and growth prove the adsorption of CCM on the surface of the working electrode.

### 3.4. Optimization of DPAdSV Parameters

**Figure 6:**
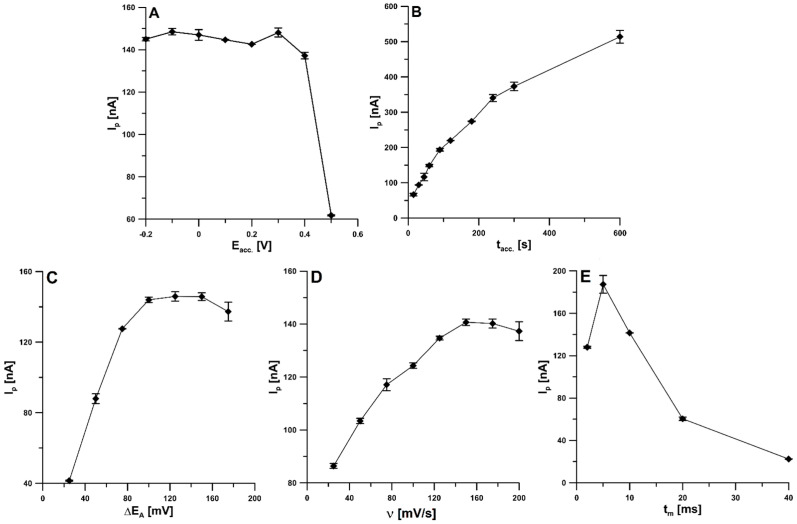
The influence of E_acc._ (**A**) and t_acc._ (**B**); ΔE_A_ (**C**); ν (**D**); and t_m_ (**E**) on 1.0 × 10^−9^ mol/L CCM signals. The average values of the CCM peak current are presented with the standard deviation of n = 3.

The effect of the accumulation potential (E_acc._) on the efficiency of the CCM adsorption process on the aSPBDDE surface was examined in the range from −0.2 to 0.5 V. The 1.0 × 10^−9^ mol/L CCM signal changes slightly when the potential changes from −0.2 to 0.3 and then decreases for more positive potentials (Figure 6A). It was decided to conduct the accumulation step at the potential of 0.3 V to oxidize compounds that could deposit on the surface of the working electrode and interfere with the analyte signal. Then, the effect of the accumulation time (t_acc._) was investigated by extending it from 15 to 600 s. The CCM analytical signal increased continuously up to 600 s (Figure 6B), but to make the analysis less time-consuming, an accumulation time of 90 s was chosen for further research.

It was also checked how the height of the CCM peak is affected by the parameters of the signal recording technique, such as amplitude (ΔE_A_), scan rate (ν), and modulation time (t_m_). The amplitude was changed from 25 to 175 mV, and it was noticed that the peak current reached the highest value at the ΔE_A_ of 100 mV; therefore that amplitude was chosen for further studies (Figure 6C). Then, the scan rate was optimized by evaluating its effect on the CCM signal in the range of 25 to 200 mV/s (Figure 6D), and it was decided that the ν of 150 mV/s would be the most suitable. Lastly, the modulation time effect in the range of 2–40 ms was tested (Figure 6E), and t_m_ of 10 ms was selected for further testing; despite the lower peak current for 10 ms than for 5 ms, for t_m_ of 10 ms, the signal was characterized by better repeatability and shape.

### 3.5. Analytical Characteristics

**Figure 7:**
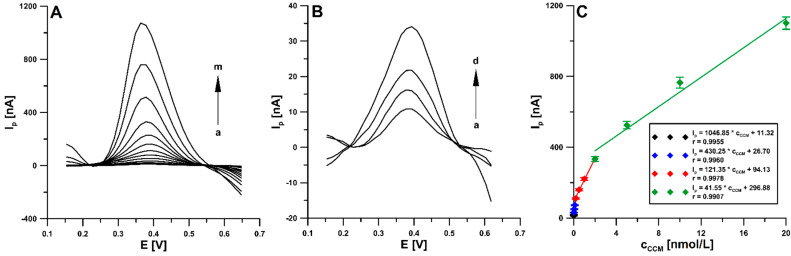
(**A**) The DPAdSVs obtained during determination of increasing CCM concentration (a → m, 2.0 × 10^−12^–2.0 × 10^−8^ mol/L) in 0.025 mol/L PBS of pH 2.6. (**B**) DPAdSV curves for CCM concentrations: 2.0 × 10^−12^ (a), 5.0 × 10^−12^ (b), 1.0 × 10^−11^ (c), 2.0 × 10^−11^, and (d) mol/L. (**C**) Calibration graph of CCM. The obtained average values of the I_p_ are shown with standard deviation for n = 3. The DPAdSV parameters: ν of 150 mV/s, t_m_ of 10 ms, ΔE_A_ of 100 mV, E_acc._ of 0.3 V, and t_acc._ of 90 s.

Under the conditions of properly selected parameters of the DPAdSV procedure, signals were recorded for successive CCM concentrations increasing linearly in the four ranges from 2 × 10^−12^ to 2 × 10^−11^, from 2 × 10^−11^ to 2 ×10^−10^, from 2 × 10^−10^ to 2 × 10^−9^, and from 2 × 10^−9^ to 2 × 10^−8^ mol/L (Figure 7). The limits of detection (LOD) and quantification (LOQ) were calculated as LOD = 3SD_a_/b and LOQ = 10SD_a_/b [35], which were 5.0 × 10^−13^ and 1.7 × 10^−12^ mol L^−1^, respectively. Numerous voltammetric procedures for the determination of CCM [1,2,3,4,5,15,16,17,18,19] can be found in the literature, but they only allow obtaining LODs of the order of 10^−8^−10^−10^ mol/L. There is only one procedure [20] in which the LOD was slightly lower (2.0 × 10^−13^ mol/L) than that obtained using an aSPBDDE, but it requires a complicated, multi-stage, and time-consuming preparation of the working electrode modifier. Moreover, the LOD is 8 to 3 orders of magnitude better than those obtained using other analytical methods for the analysis of CCM in food samples [8,9,10,11] (Table 1). It is also worth emphasizing that the proposed procedure is the first involving the screen-printed sensor in CCM analysis.

The influence of potential interferents was tested for both inorganic ions and organic compounds; the limit of tolerance was considered to be peak current changes not exceeding 10%. Based on the obtained results, it was concluded that a 1000-fold excess of Ca(II) and Mg(II) as well as glucose and ascorbic acid have a negligible effect on the 5.0 × 10^−10^ mol/L CCM analytical signal.

Moreover, the repeatability of the signal of 1.0 × 10^−9^ mol/L CCM on the aSPBDDE was checked. The relative standard deviation (RSD) of 4.97% (n = 10) was received, which confirms satisfying the repeatability of the CCM signal. Reproducibility was determined based on the results of measurements made for the same CCM concentration on three sensors. The obtained RSD value was 3.85%, which indicates good reproducibility of the aSPBDDE.

### 3.6. Analytical Application

**Figure 8:**
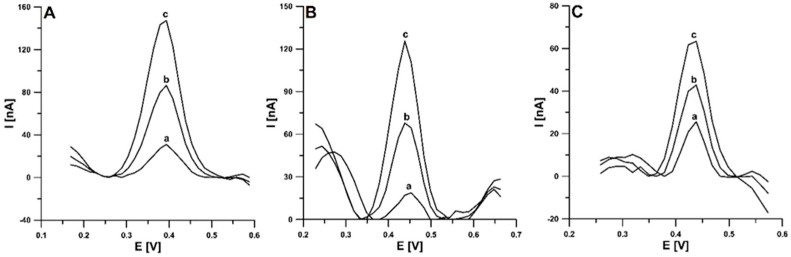
The DPAdSVs recorded during determination of CCM in turmeric extract (**A**), herbal tea infusion (**B**), and immune shot (**C**). (**A**): (a) 0.5 µL of sample (diluted with ethanol 1:99), (b) as (a) + 5.0 × 10^−10^, (c) as (a) + 1.0 × 10^−9^ mol/L CCM; (**B**): (a) 10 µL of sample, (b) as (a) + 5.0 × 10^−10^, (c) as (a) + 1.0 × 10^−9^ mol/L CCM; (**C**): (a) 2.0 µL of sample, (b) as (a) + 5.0 × 10^−10^, (c) as (a) + 1.0 × 10^−9^ mol/L CCM. The DPAdSV parameters: ν of 150 mV/s, t_m_ of 10 ms, ΔE_A_ of 100 mV, E_acc._ of 0.3 V, and t_acc._ of 90 s.

The application of the proposed voltammetric procedure was tested for the determination of CCM in food samples, i.e., turmeric extract, herbal tea infusion, and an immune shot. The voltammograms obtained during the analysis are depicted in Figure 8. Due to the low LOD allowed by the aSPBDDE, high dilutions of the analyzed samples were possible (2,000,000-fold dilution of extract, 1000-fold dilution of tea infusion, and 5000-fold dilution of shot), thus minimizing interference. The results obtained using DPAdSV were compared with the results of chromatographic analyses using ultra-high-performance liquid chromatography coupled with mass spectrometry and electrospray ionization (UHPLC-ESI/MS) (Table 2). As can be seen, the relative error values between 0.7 and 8.82% indicate a satisfactory agreement between the results obtained with the proposed procedure and comparative method and proves its usefulness in the analysis of CCM content in food samples. Moreover, the low values of the standard deviation (SD) and coefficient of variation indicate good reproducibility of the CCM signals.

## 4. Conclusions

Polyphenolic antioxidant curcumin (CCM) has a wide range of biological actions and pharmacological effects. CCM is also characterized by anti-inflammatory, anti-cancer, antimicrobial, and immunomodulatory effects. In this study, a very fast and simple voltammetric procedure using an electrochemically activated screen-printed boron-doped diamond electrode (aSPBDDE) for sensitive determination of curcumin (CCM) was proposed. The activation of the SPBDDE was performed in a solution of 0.1 mol/L NaOH by performing five cyclic voltammetric scans in the range of 0–2 V, at ν of 100 mV/s. The changes in surface morphology and reduction of the charge transfer resistance due to the activation of the electrode resulted in the amplification of the CCM analytical signal on the aSPBDDE. The registration of the differential capacity–potential curves of the double layer interface aSPBDDE/PBS of pH = 2.6 showed that the CCM electro-oxidation process on the surface of the working electrode is controlled by adsorption. The developed procedure is characterized by good selectivity and high sensitivity, allowing the achievement of a very low limit of detection (5.0 × 10^−13^ mol/L) with a wide range of linearity from 2.0 × 10^−12^ to 2.0 × 10^−8^ mol/L. The usefulness of the DPAdSV procedure was confirmed by successfully determining CCM content in food product samples. The results are in agreement with those obtained using the ultra-high-performance liquid chromatography coupled with mass spectrometry and electrospray ionization (UHPLC-ESI/MS). The results of this study allow us to consider the aSPBDDE as a very effective tool for the direct determination of CCM in food products. It should be added that the aSPBDDE is the first screen-printed sensor used to quantify CCM.

## Figures and Tables

**Table 1:** Comparison of techniques for analysis of CCM.

Method	Linear Range [mol/L]	LOD [mol/L]	Application	Ref.
CE	1.1 × 10^−3^–4.3 × 10^−3^	2.7 × 10^−5^	Plant material (*Curcuma*)	[8]
Spectrofluorimetry	1.0 × 10^−8^–1.2 × 10^−4^	9.0 × 10^−10^	Spices	[9]
Spectrophotometry	0–4.1 × 10^−5^	2.1 × 10^−7^	Spices	[10]
HPLC	6.8 × 10^−6^–8.1 × 10^−5^	1.1 × 10^−6^	Plant extracts	[11]
LC-MS/MS	6.8 × 10^−9^–1.4 × 10^−6^	-	Human plasma	[13]
RLS	1.1 × 10^−6^–1.6 × 10^−4^	1.9 × 10^−7^	Human urine	[12]
SWV	1.0 × 10^−12^–1.0 × 10^−10^	2.0 × 10^−13^	Human plasma	[14]

**Table 2:** The results of CCM determination in food product samples.

Sample	Content Determined by DPAdSV ± SD (n = 3)	Content Determined by UHPLC-ESI/MS ± SD (n = 3)	Coefficient of Variation * [%]	Relative Error ** [%]
Turmeric extract	20.97 ± 0.330 [mg/g]	19.27 ± 0.480 [mg/g]	1.6	8.8
Herbal tea infusion	14.32 ± 0.250 [µg/bag]	14.22 ± 2.51 [µg/bag]	1.8	0.70
Immune shot	175.6 ± 2.21 [µg/100 mL]	177.2 ± 11.8 [µg/100 mL]	1.3	0.90

* Coefficient of variation [%] = (SD × 100)/Content determined by DPAdSV; ** Relative error [%] = (|Content determined chromatographically—Content determined by DPAdSV|)/Content determined chromatographically) × 100.

## Data Availability

Not applicable.
